# Autologous tumor-derived heat-shock protein peptide complex-96 (HSPPC-96) in patients with metastatic melanoma

**DOI:** 10.1186/1479-5876-8-9

**Published:** 2010-01-29

**Authors:** Omar Eton, Merrick I Ross, Mary Jo East, Paul F Mansfield, Nicholas Papadopoulos, Julie A Ellerhorst, Agop Y Bedikian, Jeffrey E Lee

**Affiliations:** 1 Department of Melanoma Medical Oncology, , The University of Texas MD Anderson Cancer Center, 1515 Holcombe Boulevard, Houston, Texas, USA; 2 Department of Surgical Oncology, The University of Texas MD Anderson Cancer Center, 1515 Holcombe Boulevard, Houston, Texas, USA; 3 Department of Experimental Therapeutics, The University of Texas MD Anderson Cancer Center, 1515 Holcombe Boulevard, Houston, Texas, USA

## Abstract

**Background:**

Glycoprotein-96, a non-polymorphic heat-shock protein, associates with intracellular peptides. Autologous tumor-derived heat shock protein-peptide complex 96 (HSPPC-96) can elicit potent tumor-specific T cell responses and protective immunity in animal models. We sought to investigate the feasibility, safety, and antitumor activity of HSPPC-96 vaccines prepared from tumor specimens of patients with metastatic melanoma.

**Methods:**

Patients with a Karnofsky Performance Status >70% and stage III or stage IV melanoma had to have a metastasis >3 cm in diameter resectable as part of routine clinical management. HSPPC-96 tumor-derived vaccines were prepared in one of three dose levels (2.5, 25, or 100 μg/dose) and administered as an intradermal injection weekly for 4 consecutive weeks. In vivo induction of immunity was evaluated using delayed-type hypersensitivity (DTH) to HSPPC-96, irradiated tumor, and dinitrochlorobenzene (DNCB). The γ-interferon (IFNγ) ELISPOT assay was used to measure induction of a peripheral blood mononuclear cell response against autologous tumor cells at baseline and at the beginning of weeks 3, 4, and 8.

**Results:**

Among 36 patients enrolled, 72% had stage IV melanoma and 83% had received prior systemic therapy. The smallest tumor specimen from which HSPPC-96 was prepared weighed 2 g. Twelve patients (including 9 with stage IV and indicator lesions) had a negative DNCB skin test result at baseline. All 36 patients were treated and evaluable for toxicity and response. There were no serious toxicities. There were no observed DTH responses to HSPPC-96 or to autologous tumor cells before or during treatment. The IFNγ-producing cell count rose modestly in 5 of 26 patients and returned to baseline by week 8, with no discernible association with HSPPC-96 dosing or clinical parameters. There were no objective responses among 16 patients with stage IV disease and indicator lesions. Among 20 patients treated in the adjuvant setting, 11 with stage IV melanoma at baseline had a progression-free and overall survival of 45% and 82%, respectively, with a median follow-up of 10 years.

**Conclusion:**

Treatment with autologous tumor-derived HSPPC-96 was feasible and safe at all doses tested. Observed immunological effects and antitumor activity were modest, precluding selection of a biologically active dose. Nevertheless, the 25-μg dose level was shown to be practical for further study.

## Introduction

The past two decades have witnessed increasingly sophisticated approaches to incorporating active immunotherapy into the multimodality care of the population of oncology patients for whom there continues to be significant unmet medical need. This field of active immunotherapy has been challenged by an evolving understanding of the complexity of host-tumor interactions, a lack of availability of clinical grade tests to confirm the induction of antitumor immunity in the systemic circulation or in tissue compartments, and the need to overcome biophysical and other barriers to effective tumor eradication. Increasingly sensitive measures of systemic cellular immune response, such as the γ-interferon (IFNγ) enzyme-linked immunospot (ELISPOT) and tetramer assays, may facilitate the early clinical development of cancer vaccines.

Gp96 is a non-polymorphic constitutively expressed and inducible heat-shock protein (HSP) which associates with intracellular antigenic peptides. Such gp96-peptide complexes have been shown to elicit potent tumor-specific T cell responses and protective immunity in a variety of animal models. For example, immunotherapy of mice with preexisting cancer (including spontaneously derived B16 melanoma) treated with HSP preparations derived from syngeneic cancer resulted in a delay of progression of the primary cancer, a reduced metastatic load, and prolongation of life span, whereas treatment with HSP preparations derived from cancers other than the syngeneic cancer did not provide such protection [1,2]. These studies were especially interesting in that they showed an autologous antitumor response without identifying the specific tumor antigenic epitopes [1]. Furthermore, HSP-96 peptide complex (HSPPC-96; Vitespen, formerly Oncophage) was shown to elicit antigen-specific cytotoxic T lymphocytes (CTLs), whereas gp96 alone, peptide alone, or Freund's adjuvant with peptide did not elicit such antigen-specific CTLs [2].

Srivastava et al. proposed a peptide relay model to explain these findings wherein HSPs shuttle peptides from the proteosome to the endoplasmic reticulum; in the endoplasmic reticulum, immunogenic peptides bound to HSP-96 may transfer to the major histocompatibility complex (MHC). Supporting this model, HSP-96 and MHC have homology in the peptide-binding domains [3].

Many peptides being evaluated in melanoma immunotherapy trials are restricted in binding to a specific human MHC haplotype (human leukocyte antigen [HLA].) for presentation on the cell surface. However, gp96 is non-polymorphic; thus, gp96-derived vaccines could potentially have broader applicability than HLA-restricted peptide vaccines. Immunizing mice of the H-2^b ^haplotype with HSPPC-96 from SV40-transformed cells of the H-2k haplotype resulted in an H-2b-restricted antigen-specific CTL response [3]. Suto and Srivastava demonstrated that exogenous viral peptides chaperoned by gp96 could be channeled into the endogenous pathway of specialized macrophages and presented through MHC class I molecules, resulting in CD8+ CTL activation [4]. These findings supported a structural basis for cross-priming: specialized professional antigen-presenting cells (APCs; e.g., macrophages and dendritic cells) from the immunized mice could salvage HSPPC-96 from damaged cells and present it in the context of MHC class I molecules, ultimately resulting in an endogenous CTL immune response. In support of this idea, CD91 was identified as the HSPPC-96 receptor on these APCs [5]. Thus, treatment with tumor-derived HSPPC-96 presumably could provide an array of autologous tumor-specific peptide targets (and even targets from endothelial and other cells in the metastases) for CTL activation, all without the need to characterize each peptide or exclude patients on the basis of HLA phenotype.

Toxicology studies in mice treated with multiple doses (0-100 ng) of HSPPC-96 over the course of either 2 or 4 weeks revealed no adverse consequences on body weight or general health. Lymphoid hyperplasia was noted in some mice. Notably, metastases in the treated mice were smaller than those in control tumor-bearing mice, and survival was longer [1]. One limitation of transitioning this treatment to patients was that it was unknown whether sufficient HSPPC-96 could be purified from resected metastases to provide adequate doses. Another limitation was the challenge of collecting, preparing, standardizing, and certifying biologic material for treatment derived from individual patients' tumors. Furthermore, at the time this study was conducted, HSPPC-96 had been shown to be stable for only up to 2 months from the time of preparation, precluding treatment for longer periods. HSPPC-96 has since been shown to be stable for longer periods [6].

HSPPC-96 was first evaluated in humans in a small trial in Germany in advanced cancer patients. Janetzki *et al *showed that immunization with 25 μg of HSPPC-96 elicited MHC Class I-restricted, tumor-specific CD8+ T lymphocytes in 6 of 12 patients with advanced cancer using the IFNγ ELISPOT assay [7]. To determine the utility of HSPPC-96 as a treatment for melanoma, we undertook the first feasibility trial in the United States in 1997. The goals of this study were to (1) evaluate the feasibility of vaccine preparation, (2) determine the safety and tolerance of 2.5, 25 or 100 μg/dose of HSPPC-96 administered by the intradermal route weekly for 4 weeks, (3) detect induction of a tumor-specific immune response against autologous tumor, and (4) document any observed antitumor activity. The three dose levels were chosen empirically based on the predicted yield from a minimum of 2 g of tumor. The schedule was limited to only 4 injections over 4 weeks. Detecting the induction of a cellular immune response against autologous tumor in a reproducible manner would provide justification for the clinical development of HSPPC-96 as an anticancer agent.

## Methods

### Patients

Patients evaluated at M.D. Anderson were required to have clinically confirmed advanced regional (nodal or in-transit) melanoma (stage III) or distant metastases (stage IV) and Karnofsky Performance Status scores >70%. Prior systemic treatment with chemotherapy drugs or cytokines was permissible. Patients undergoing resection of large (>3 cm) histologically confirmed metastatic melanoma as part of their routine clinical management and who agreed to participate in the study signed an Institutional Review Board (IRB)-approved consent form for procurement of tissue for autologous tumor-derived HSPPC-96 preparation. The resected metastasis needed to yield ≥ 5 g of non-necrotic tumor so that we could perform routine clinical pathologic study, vaccine preparation, and treatment-related bioassays. Four weeks after tumor resection, the patients had to be fully recovered from surgery and to demonstrate a <25% increase in known visceral metastases and no appearance of new metastases in liver, bone, or brain on follow-up staging procedures. Patients were then required to sign a second IRB-approved consent form for HSPPC-96 treatment.

Ineligibility criteria included: pregnancy; severe intercurrent illness; routine use of steroids, non-steroidal anti-inflammatory agents, or H2 antagonists; granulocyte count <1,500/mm^3^, platelet count <90,000/mm^3^; serum creatinine level >1.5 mg/dl; or bilirubin level >1.2 mg/dl. All indicator lesions were documented using physical examination, computed tomography, magnetic resonance imaging, and, for skin lesions, photography just before informed consent was signed for treatment. Patients underwent baseline black-light examinations to detect the presence of vitiligo.

Patients recorded symptoms in a patient diary, and adverse effects were monitored weekly and graded using World Health Organization (WHO) criteria [8]. A physical examination was performed at weeks 4 and 8. Patients with any vision complaints were referred to an ophthalmologist for evaluation. Subsequently, history, physical examination, and laboratory and radiologic testing were performed every 6-8 weeks until evidence of progressive disease or for the first 6 months. After 6 months, the formal staging interval reverted to current practice guidelines and was dependent on stage and extent of disease. Objective tumor responses were evaluated using WHO criteria [8].

### Tumor Procurement and Initial Processing

A protocol specialist assisted the surgical pathologist in dissecting tumor specimens immediately on delivery from the operating room in a sterile wrap on ice. After a small tumor specimen was set aside for clinical pathological review, the bulk of each tumor specimen (minimum of 2 g of fresh non-necrotic tumor tissue) was used for vaccine preparation and was dissected, placed into a labeled 50-ml pyrogen-free vial in a plastic zip-lock bag on dry ice, and sent in a polystyrene box with a temperature monitor by overnight air delivery to the Antigenics Inc. vaccine preparation facility in Framingham, MA. In exceptional cases, samples procured after hours, on holiday, or over the weekend were stored in a -70°C freezer, to be held for shipping on the next business day. Residual non-necrotic tumor (minimum 1-2 g) was processed immediately on site for *in vivo *human delayed-type hypersensitivity (DTH) assays and for specialized *in vitro *immunologic assays (see below).

### **Autologous Tumor-Derived HSPPC-96 Preparation**

Details of HSPPC-96 preparation are available elsewhere [6,9]. Briefly, at the Current Good Manufacturing Practice (CGMP) certified facility in Framingham, MA, the tumor specimens were thawed, minced, suspended in sodium bicarbonate (pH 7.0), and homogenized. The homogenate was centrifuged and protein from the supernatant was selectively precipitated by a two-step ammonium sulfate precipitation at 50% and 80% saturation levels, followed by affinity chromatography on Con-A Sepharose and ion-exchange chromatography using a Diethylamino Ethanol (DEAE) column. HSPPC-96 was eluted and tested for purity, homogeneity, and identity by SDS-PAGE and Western blotting. Buffer exchange was performed to isotonic saline, and the vaccine was sterile filtered, aliquoted, and stored at -80°C. The concentration of each individual's HSPPC-96 was given as microgram per milliliter. The release criteria for each patient's vaccine included: 1) ≥ 50% 96-kDa band by SDS-PAGE gel; 2) sterility by USP sterility test; 3) minimal endotoxin content by *Limulus *amoebocyte assay. The dose level for each patient was determined by the amount of vaccine material available for four equal aliquots of 2.5, 25, or 100 μg each. The full vaccine series for each patient was returned by overnight mail in four individual vials on dry ice to the M. D. Anderson Investigational Drug Pharmacy, where the vials were stored in a -70°C freezer until the patient was ready for treatment.

### Autologous Tumor-Derived HSPPC-96 Administration

In the Melanoma Medical Oncology Clinic at M. D. Anderson, a vial of HSPPC-96 was thawed and the contents drawn up into a tuberculin syringe and injected intradermally into either the patient's anterior deltoid, medial subinguinal, or subclavicular region. Areas distal to surgically affected lymph node basins were avoided. Ten patients were to be treated at each of three dose levels of HSPPC-96 (2.5, 25, or 100 μg). Treatment was administered weekly for 4 consecutive weeks. Patients could be retreated with HSPPC-96 from a second harvested tumor.

### Immunological Monitoring

#### Skin Testing

Prior to weekly vaccines 1 (baseline) and 4 (beginning of week 4) and at the first month follow-up visit after dose 4 (week 8), the research laboratory provided two insulin syringes for DTH control assays using intra-dermal injection on the volar surface of the forearms. One syringe contained confirmed sterile 10^5 ^mechanically dissociated X-irradiated (20 Gy) autologous tumor cells from the original surgical specimen cryopreserved in 10% dimethylsulfoxide and 10% human albumin in saline. The other syringe contained 10^5 ^X-irradiated autologous peripheral blood leukocytes serving as a negative control, cryopreserved in a similar manner.

Because the treatment route for HSPPC-96 was in the skin, delayed cutaneous hypersensitivity to 2,4-dinitrochlorobenzene (DNCB) was used to test *de novo *immunity to this chemical, based on assays developed and tested in cancer and melanoma patients since the 1960s [11,12]. After cleaning the skin with acetone, 2000 and 50 μg of DNCB in 100 μl of acetone were layered on the skin of the volar aspect of the forearm, and each dose was confined by a ring with 2 cm inner diameter. After drying with portable hair dryer, each site was covered with a bandage for 24 hours, resulting in an erythematous reaction that cleared in a few days. Between 9 and 21 days, induration at both sites confirmed a grade 4 DTH response and induration at the 2000 μg site only indicated a grade 3 DTH response. With no response at either site, retesting with 50 μg, yielding a >5 mm induration after 48 hours confirmed a grade 2 DTH response. No effort was made to distinguish a grade 1 from a grade 0 response, because this would have required a skin biopsy [10-12].

#### Peripheral Blood Mononuclear Cell (PBMC) Assays

For PBMC assay, 40 ml of peripheral blood was collected into heparinized Vacutainer tubes prior to the first, third, and fourth treatment with HSPPC-96 and at the 8-week follow-up visit. PBMCs were isolated by density-gradient separation (using Histopaque^®^-1077; Sigma-Aldrich, St. Louis, MO) and cryopreserved at -130°C in a solution of 90% human AB serum + 10% dimethylsulfoxide. Tumors were dissociated by enzymatic digestion, and the cells were enriched by fractionation on a 2-step gradient of 75% and 100% Histopaque^® ^prior to cryopreservation. On the day of testing, PBMCs from each of the four collection days were rapidly thawed at 37°C, serially diluted and washed with warm RPMI 1640 supplemented with 10% fetal bovine serum (FBS), HEPES buffer, glutamine, and antibiotics (supplemented RPMI [S-RPMI].), then adjusted to a concentration of 1.5 × 10^6^/ml in S-RPMI. Cryopreserved autologous tumor cells were also thawed, and, unless otherwise indicated, depleted of tumor-infiltrating leukocytes by immunomagnetic removal of CD45^+ ^cells (Miltenyi Biotech, Auburn, CA). The tumor cells were then adjusted to 7.5 × 10^5^/ml in S-RPMI.

An ELISPOT assay was used to analyze the effect of HSPPC-96 treatment on the frequency of IFNγ-secreting cells in peripheral blood. The IFNγ ELISPOT assay has been reported to be a good indicator of the presence of CTL [13], and CD8^+ ^MHC class I-dependent IFNγ-secreting cells have been detected in patients [14]. A matched pair of monoclonal anti-human IFNγ capture and biotinylated anti-IFNγ detection antibodies were obtained from Endogen (Woburn, MA). Briefly, 96-well nitrocellulose-backed plates (Millipore, Bedford, MA) were coated overnight with anti-human IFNγ capture antibodies (10 μg/ml solution of antibody in phosphate-buffered saline [PBS].). After washing, the plates were blocked with PBS containing 10% FBS. PBMCs and tumor cells were then added in equal 100-μl volumes to replicate wells (2:1 PBMC:tumor ratio). Additional test/control wells included unstimulated PBMCs cultured in medium alone, tumor cells cultured in medium alone, and PBMCs cultured with anti-CD3 antibody as a polyclonal stimulator (OKT3; Ortho Biotech Inc., Raritan, NJ, diluted to 1 μg/ml in S-RPMI).

PBMCs from one or two healthy donors without cancer were also tested for IFNγ production in response to the same stimuli. The normal donors served as a positive control for the assay conditions (positive response to anti-CD3) and provided information with regard to the integrity and stimulatory capabilities of the tumor cells (IFNγ release from normal lymphocytes in response to the allogeneic stimulus). The plates were incubated for 40 hours at 37°C in a 5% CO_2 _humidified atmosphere. After incubation, the plates were washed 4 times with PBS and 4 times with Tween/PBS (0.025% Tween 20 diluted in PBS). Biotinylated detection antibody (1 μg/ml solution in PBS + 4% bovine serum albumin) was added to each well, and the plates were incubated at room temperature for 1 hour. The plates were then washed again with Tween/PBS. Streptavidin peroxidase (Zymed Laboratories Inc., San Francisco, CA, 1:1000 dilution in Tween/PBS) was added to each well, and the plates were incubated for another 30 min at room temperature. After four additional washes with Tween/PBS, AEC substrate (Sigma) was added for approximately 5 min to develop the plates. Finally, the plates were washed with tap water, dried, and the number of spots, each coinciding to a single cytokine-producing cell, was counted under a dissecting microscope.

We emphasize that PBMCs were not stimulated or expanded in culture other than as specified above during the ELISPOT assay. The mean values of spots in replicate wells were determined, and the frequency of IFNγ-secreting cells in tumor-stimulated PBMCs is reported as the number of spots per 1.5 × 10^5 ^PBMCs after subtraction of controls. In the case of PBMC-tumor mixtures, controls consisted of the average number of spots produced by unstimulated PBMCs plus the average number of spots in wells that contained tumor cells alone.

**Figure 1:**
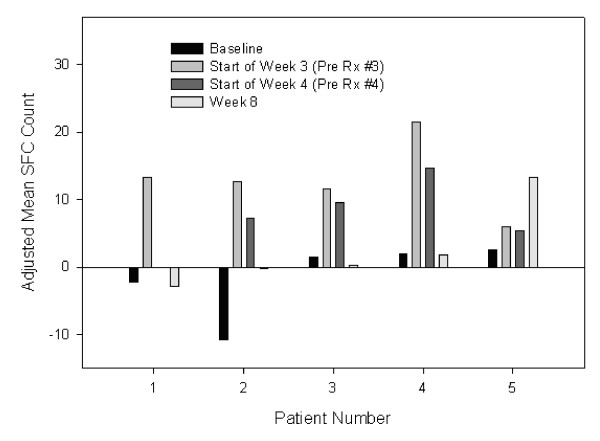
**Mean number of spot forming peripheral blood mononuclear cells producing γ-interferon (SFC) in the presence of autologous tumor cells, corrected for mean number of SFC in the absence of tumor cells, using the γ-interferon ELISPOT assay**. Rx refers to the HSPPC-96 vaccine dose.

The number of spot-forming cells (SFCs) corresponding to IFNγ-producing PBMCs cultured 2:1 with autologous tumor cells was recorded in each well loaded with 1.5 × 10^5 ^PBMCs. Measurements were recorded in duplicate or triplicate. From the mean SFC count was subtracted the mean number of SFCs caused by IFNγ-producing PBMCs in the absence of tumor cells and caused by tumor in the absence of PBMCs. The latter control value could be other than zero if the tumor cells were contaminated by tumor-infiltrating lymphocytes. Such background IFNγ ELISPOT activity was not observed in tumors depleted of CD45^+ ^leukocytes prior to testing in the ELISPOT assay but was observed when sparse tumor cell samples precluded optimal immunosorting (4 cases). As shown in Figure 1 for patient 1, this background could exceed the number of SFCs detected in the baseline tumor-stimulated PBMCs, resulting in a negative adjusted SFC count. Alternatively, the adjusted SFC count could turn out to be negative when tumor cells actively suppress PBMC IFNγ production as in patient 2 wherein SFC detected in the absence of tumor stimulators were quenched by the addition of autologous tumor cells.

### Statistical Considerations

Three dose levels spanning 2.5-100 μl were chosen on the basis of the first feasibility study in patients in Germany [7]. This dosing range, although narrow, could provide evidence of a biologically active lowest dose, which could facilitate a broader clinical development strategy. To be evaluable, a patient was required to complete 4 weekly treatments with HSPPC-96 and the first post-treatment follow-up evaluation at 8 weeks. It was anticipated that at least 15% of patients registered would not be evaluable for biological response because of technical difficulties with the assays. This aims of this pilot study were in order: feasibility, safety, and detection of an immunological response against autologous tumor by DTH or ELISPOT assays described earlier. Any evidence of immunological or clinical response would support further development in phase 2 studies.

## Results

Between January 1998 and October 1999, 58 patients signed informed consent for tumor procurement and underwent surgical resection of metastases. Six additional patients were accrued on this trial (in addition to the 52 originally planned) as a result of trying to fill the 100 μg cohort, which ultimately remained undersubscribed by one patient.

Clinical-grade HSPPC-96 was successfully prepared from 96% of tumor specimens, some of which weighed only 2 gm. Two specimens were inadequate for HSPPC-96 preparation because of excessive necrotic tumor in one specimen and excessive melanin impeding vaccine preparation in another. Thirty-six (62%) of 58 patients were treated with autologous tumor-derived HSPPC-96. Twenty patients for whom HSPPC-96 was available received alternative treatment, mostly as a result of ineligibility resulting from early progression of disease (see above).

The clinical characteristics of the 36 patients who received HSPPC-96 are listed in Table 1. The Karnofsky performance status of all patients was 80%-90%. All but 6 patients had received prior systemic therapy for melanoma, and 27 (75%) had previously received the cytokines IFNα2 or interleukin-2 (IL2) alone or in combination with chemotherapy. All patients had normal baseline serum levels of both lactate dehydrogenase (LDH) and albumin.

**Table 1:** Patient Characteristics

HSPPC-96 Dose Level	Total	2.5 μg	25 μg	100 μg
Enrolled, no.	36	11	16	9
Male, no (%)	26 (72%)	8	11	7
Median age (range), years	54 (16-75)	53	53	56
Karnofsky performance status				
90%	18	5	9	4
80%	18	6	7	5

Prior treatment: no. of regimens				
0	6		2	4
1	19	8	8	3
2	4	1	1	2
≥ 3	7	2	5	
Prior treatment: type				
IFN-α2 alone	11	7	3	1
IL-2 alone	2	1	1	
IFN + IL-2	2	1	1	
Chemotherapy + IFN + IL-2	18	4	10	4
Chemotherapy + IFN	2		2	
Systemic chemotherapy alone	14	4	9	1

Elevated serum LDH level	0	0	0	0
Serum level albumin <3.4 mg/dl	0	0	0	0

**Melanoma Characteristics**				
Regional nodal disease alone	6 (17%)	2	2	2
Regional nodal and in-transit disease	5 (14%)	2	3	
Advanced disease	25 (69%)	7	11	7
No. of visceral organs involved				
0 (subcutaneous, nodal)	5	2	1	2
1	14	2	7	5
2	6	4	2	
3	1		1	
Visceral sites of disease				
Lung alone	10	1	4	5
Gastrointestinal tract alone	2		2	
Brain alone	2	1	1	
Lung + 1 other visceral organ	5	3	2	
Liver + 1 other visceral organ	3	3		
Brain + 1-2 other visceral organs	2		2	

**HSPPC-96 derivation**				
Subcutaneous metastases	7	1	6	
Lymph node metastases	19	7	7	5
Lung metastases	7	1	2	4
Liver or GI metastases	3	2	1	

**HSPPC-96 treatment setting**				
**Indicators**	**16 (44%)**	**6**	**7**	**3**
Stage III disease	2		2	
Stage IV disease	14	6	5	3
**Adjuvant**	**20 (56%)**	**5**	**9**	**6**
Stage III disease	9	4	3	2
Stage IV disease	11	1	6	4

Ten patients had evidence of regional metastatic disease in lymph nodes or subcutaneous tissue at the time of HSPPC-96 treatment. Twenty patients were treated in the adjuvant setting (56%). Among 26 patients with stage IV melanoma, a median of one visceral organ involved (range, 0-3), with 10 patients having only lung metastases.

### Toxicities

Adverse events are presented in Table 2. There were no WHO or Common Terminology Criteria for Adverse Events (CTCAE) Version 3.0 grade ≥ 3 toxicities reported and no toxicities definitively attributable to the 4 weekly treatments with HSPPC-96. One patient who received 25 μg and 2 patients who received 100 μg reported fleeting nonspecific vision changes (blurry vision); in all three cases, formal ophthalmologic evaluations proved unrevealing and vision was objectively normal. A patient who received 100 μg developed a herpes zoster reactivation concurrent with progressive melanoma 1 week after treatment with HSPPC-96.

**Table 2:** Adverse Events by Dose Level

Grade 1 Adverse Event	N (%)	2.5 μg	25 μg	100 μg
Number of patients	36	11	16	9
Nausea	8 (22)	2 (18)	3 (19)	3 (33)
Fatigue	7 (19)	3 (27)	2* (12)	2 (22)
Headache	7 (19)	3 (27)	2 (12)	2 (22)
Constipation	5 (14)	2 (18)	2 (12)	1 (11)
Asthenia	4 (11)	1 (9)	1 (6)	2 (22)
Pyrexia	4 (11)	1 (9)	1 (6)	1 (11)
Visual change	3 (8)		1** (6)	2** (12)
Zoster reactivation	1 (3)			1 (11)

* Grade 2, one patient

** Fleeting, not associated with abnormal ophthalmologic exam

In the 25 μg dose group, a 47-year-old patient developed symmetric punctuate vitiligo around his neck (not involving the site of his resected primary melanoma, which was on his thigh), approximately 4 months after the start of treatment. This finding may not be directly attributable to HSPPC-96 treatment, in part because this patient had had a clinical response to biochemotherapy with IFNα2 and IL2 less than 6 months prior to the start of HSPPC-96 treatment. This patient did not have a DTH response at baseline to DNCB, suggesting cutaneous anergy. Furthermore, IFNγ ELISPOT data for this patient never rose above a low baseline mean value during HSPPC-96 treatment. Based on the surgeon's report, the patient had an incompletely resected pelvic mass; however, the residual disease was not evaluable by computed tomography scan prior to the start of HSPPC-96 treatment. The patient remained without progression of disease for 61 months but ultimately died of leptomeningeal metastases at 63 months.

### DTH Reactions

Individual patient biomarker results are summarized in Table 3, together with clinical activity data. Nine, fourteen, and one patient(s), respectively, had grade 4, 3, and 2 DTH reactions to DNCB at baseline. Twelve patients had no reaction to DNCB (grade 0-1 [33%].), including three of the patients treated in the adjuvant setting (15%) and nine treated with indicator lesions (56%). Cutaneous anergy as measured by this assay was thus more prevalent among patients with indicator lesions (p = 0.01, Fisher's exact test).

There were no clear-cut DTH responses observed to HSPPC-96 at any dose level tested. Similarly, during the 8-week period, there were no DTH responses to 10^5 ^lethally irradiated autologous tumor cells or to the peripheral blood leukocyte control administered by the subcutaneous route.

### IFNγ ELISPOT assay

Individual patient biomarker results are summarized in Table 3, together with clinical activity data. From a total of 26 patients evaluated using the IFNγ SFC assay, only 5 (19%) had a modest and transient increase in average SFC count during the 8-week study period, as summarized in Figure 1. Patients 2, 3, and 5 were given 2.5 μg, and patients 1 and 4 were given 25 μg of HSPPC-96 in weekly doses × 4. In most patients the increase in SFC count returned to baseline or near baseline levels by week 8. The most noticeable increase in SFC was observed in patients 4 and 5, both of whom had markedly rapid progression of disease, supporting the detection of a strong but clinically ineffective immune response in the course of treatment with HSPPC-96. In contrast, patients 1 and 2, who were treated in the adjuvant setting, had negative baseline SFC counts, achieved transient modest SFC elevations, and have remained free of disease for >9 years since HSPPC-96 treatment. No patient from the 100 μg group had even a transient increase in average SFC count.

**Table 3:** Individual Patient Clinical and Biomarker Data

						Delayed Type Hypersensitivity (DTH)				γ-interferon ELISPOT*							
					Dose Level	DNCB	HSPPC96	Autol. tumor	PBMC					OR	TTP	OS	
M/F	Age	# prior	KPS	Stage**	μg	Grade***				BL	Wk 3	Wk 4	Wk 8		Months		Alive
**TREATED WITH INDICATOR LESIONS PRESENT**																	
F	53	4	80	4B	2.5	4	0	n/d	n/d	-0.3	0.5	-0.3	0.5	PD	1.6	21.3	
M	36	2	80	4B	2.5	0	0	n/d	n/d	1.5	11.5	9.5	0.0	SD	1.6	35.0	
M	56	1	80	4B	2.5	4	n/d	n/d	n/d	n/d	n/d	n/d	n/d	PD	1.9	2.4	
M	56	3	90	4B	2.5	0	0	0	0	0.0	-1.7	-9.0	-2.3	PD	2.1	38.2	
M	61	1	90	4B	2.5	3	0	n/d	n	2.5	6.0	5.3	13.3	PD	1.8	18.0	
M	52	1	80	4B	2.5	0	0	0	0	n/d	n/d	n/d	n/d	PD	1.6	15.9	
F	56	1	90	3N2C	25	0	0	0	0	0.3	-0.3	-1.3	0.3	PD	1.5	6.0	
M	16	1	80	3N2C	25	0	0	0	0	2.0	21.5	14.7	1.8	PD	0.2	6.9	
M	65	5	80	4B	25	3	0	n/d	n/d	n/d	n/d	n/d	n/d	PD	1.2	11.7	
M	50	1	90	4B	25	4	0	0	0	n/d	n/d	n/d	n/d	PD	1.0	3.7	
M	47	3	80	4B	25	0	0	0	0	n/d	n/d	n/d	n/d	PD	0.7	24.6	
M	58	3	80	4B	25	4	0	0	0	0.0	0.0	0.0	0.0	PD	1.7	16.0	
M	47	2	90	4B	25	0	0	n/d	n/d	0.5	-0.3	0.2	-1.7	SD	60.6	62.7	
M	32	1	80	4A	100	0	0	0	0	n/d	n/d	n/d	n/d	PD	0.7	6.7	
M	59	0	80	4B	100	3	0	0	0	-1.3	0.0	0.0	0.0	PD	4.4	24.0	
M	76	0	80	4B	100	0	0	0	0	0.5	n/d	1.0	n/d	SD	14.6	15.5	
**TREATED IN THE ADJUVANT SETTING**																	
F	63	1	80	3N2C	2.5	3	0	0	0	0.7	2.0	1.3	1.7	PD	4.0	12.3	
M	47	1	90	3N1	2.5	3	0	n/d	n/d	0.0	1.0	1.0	0.0	NED	99.5	99.5	Alive
F	40	1	90	3N1	2.5	4	0	n/d	n/d	0.0	0.5	0.5	0.5	NED	101.1	101.1	Alive
M	64	1	80	3N2C	2.5	3	0	n/d	n/d	1.0	0.3	1.0	1.3	NED	110.5	110.5	Alive
M	52	1	90	4A	2.5	0	0	n/d	n	-10.7	12.7	7.3	0.0	NED	116.1	116.1	Alive
M	63	0	90	3N2C	25	3	0	0	0	1.0	-0.3	2.0	2.0	PD	1.6	20.4	
M	59	1	90	3N2A	25	3	0	0	0	0.0	0.0	0.0		PD	8.1	18.2	
M	63	1	90	3N2A	25	3	0	n/d	n/d	0.0	0.0	0.0	0.0	PD	26.7	30.9	
F	70	3	90	4A	25	0	0	0	0	9.0	0.0	0.5	2.5	NED	110.3	110.3	Alive
M	46	1	80	4B	25	3	n/d	n/d	n/d	n/d	n/d	n/d	n/d	PD	1.7	14.4	
F	55	1	90	4B	25	4	0	trace	0	n/d	n/d	n/d	n/d	PD	21.3	122.0	Alive
F	41	1	80	4B	25	4	0	0	0	0.0	0.3	0.0	0.0	PD	30.4	119.5	Alive
F	48	0	90	4B	25	3	0	0	0	-0.5	0.5	2.0	1.5	NED	68.8	68.8	Alive
M	44	4	80	4B	25	4	0	0	0	-2.3	13.3		-2.9	NED	116.1	116.1	Alive
M	58	0	90	3N2A	100	3	0	0	0	n/d	n/d	n/d	n/d	PD	27.6	41.9	
F	43	0	90	3N2A	100	4	0	0	0	0.0	0.5	-1.0	-0.3	NED	30.1	30.1	Alive
M	65	2	90	4A	100	3	0	0	0	0.7	0.7	-5.3	-0.3	PD	27.8	119.7	Alive
M	56	1	80	4B	100	3	0	0	0	0.7	0.3	1.3	-0.3	PD	5.4	11.3	
F	44	1	80	4B	100	0	0	0	0	n/d	n/d	n/d	n/d	PD	15.7	106.8	Alive
M	42	2	90	4B	100	2	0	0	0	14.3	8.3	8.7	14.3	NED	119.6	119.6	Alive

* Mean # spot forming PBMC producing γ-interferon (SFC) in the presence of autologous tumor cells, corrected for mean # SFC in the absence of tumor cells

** 1992 American Joint Commission on Cancer Staging System

*** For all time points (Baseline, week 4, week 8), DTH was Grade 0 for HSPPC-96, autologous tumor, PBMC in all patients tested

Abbreviations:

# prior Number prior regimens

OR Objective response

Autol. Autologous

OS Overall Survival

BL Baseline

PD Progression of Disease

KPS Karnofsky performance status

SD Stable Disease

M/F Male/Female

TTP Time to prtogression

n/d Not done

Wk Week

NED No evidence of recurrent disease (treated in the adjuvant setting)

Mean SFC counts were elevated at baseline (9 and 14.3 SFCs) in two patients with stage IV disease who were treated in the adjuvant setting; both patients had experienced progressive disease in nodal and pulmonary metastases, respectively, while receiving an IL2 containing regimen prior to enrollment in this trial. After surgical resection and treatment with HSPPC-96, both patients have since remained free of disease for >10 years. The first patients had a persistent dip in mean SFC from 9 at baseline to 0 during the vaccination period, rising to 2.5 four weeks after the last dose of HSPPC-96.

### Anti-Tumor Activity and Clinical Course

Individual patient biomarker results are summarized in Table 3, together with clinical activity data. There were no major responses (complete or partial) among 16 patients with indicator lesions (6, 7, and 3 patients given 2.5, 25, and 100 μg of HSPPC-96, respectively). A 76-year-old man given 100 μg had initial progression in a 2-cm pulmonary metastasis at 8 weeks, followed by near complete resolution of this lesion by 6 months; however, several other pulmonary nodules slowly progressed, resulting in a mixed response. The 47-year-old-patient (25 μg dose level) with an incompletely resected pelvic mass remained free of measurable disease for 61 months before disease progression, as detailed earlier (see Toxicities). A 37-year-old patient (2.5 μg dose level) had progression in a 4-cm paraaortic node at 8 weeks, but this then stabilized for 10 months before resuming progression. None of these 3 patients reacted to DNCB, and only the third (Figure 1, patient 3) had a transient increase in SFCs according to the IFNγ ELISPOT assay.

A 44-year-old patient had had lung, liver, and bone metastases which progressed on chemotherapy but responded completely to high-dose IL2 treatment. He presented with a huge burden of axillary disease with peripheral neuropathy 2 years later and underwent amputation. He was treated with HSPPC-96 derived from the axillary disease (25 μg dose level) in the adjuvant setting and has remained free of recurrence for 10 years. He had a robust (grade 4) DTH response to DNCB at baseline. He is also patient 1 in Figure 1 and had a transient increase in SFCs. Two patients with subcutaneous and lung metastases, respectively, underwent a second surgical resection and treatment with 2.5 μg of fresh HSPPC-96 and showed no evidence of clinical activity.

Two patients with stage III disease with in-transit disease had immediate progression of disease and died in 6 months. Nine patients with stage III disease treated in the adjuvant setting had a median time to progression of 28 months and median overall survival of 31 months, with 4 patients (44%) alive and without progression of disease at 10 years.

**Figure 2:**
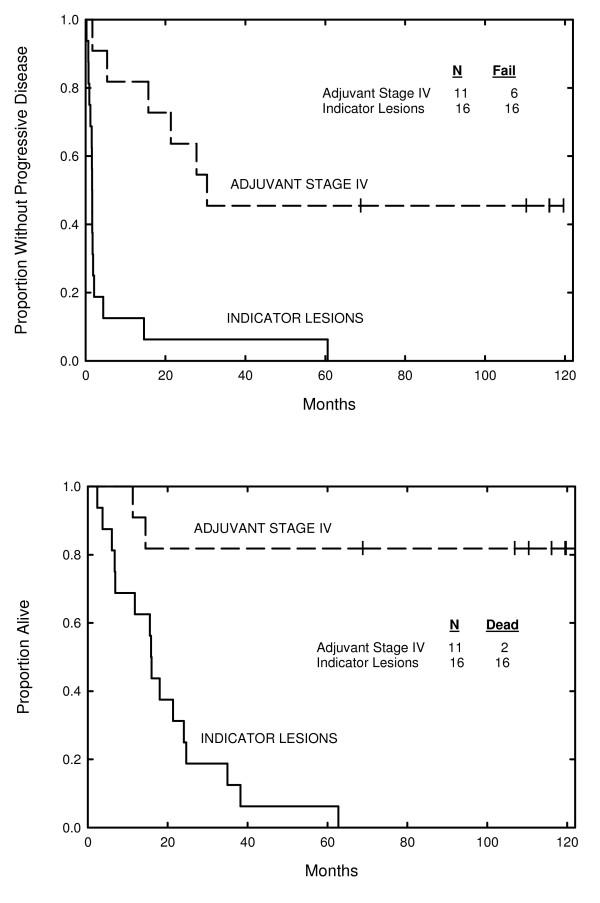
**Kaplan-Meier curves for time to disease progression (A) and overall survival (B) of patients with metastatic melanoma treated with indicator lesions (n = 16) or treated in the stage IV adjuvant setting (n = 11)**.

For patients with stage IV disease, Kaplan-Meier curves for time to progression and overall survival are presented disease in Figure 2. Among 16 patients with stage IV disease who had indicator lesions, 13 (81%) had early evidence of progression of disease at the first follow-up scan interval (6-8 weeks), and their median overall survival was 15.9 months (range, 2.4-62.7 months). In contrast, among the 11 patients with stage IV disease treated in the adjuvant setting, the median time to progression was 30.4 months (range, 1.7 to >10 years) with 9 still alive (82%) after a median follow-up period of 10 years.

## Discussion

This study confirms the feasibility of routinely acquiring, processing, and preparing clinical-grade HSPPC-96 in a timely manner from fresh tumor weighing as little as 2 g for use in patients with metastatic melanoma. The current study was limited to weekly dosing for 4 consecutive weeks by an imposed 2-month shelf life for HSPPC-96, which has since been extended [6]. Based on the experience in the 36 patients reported here, a dose of 25 μg was technically feasible for ≥ 4 consecutive treatments, whereas we had difficulty filling the 100 μg cohort (400 μg total dose). With the widespread adoption of sentinel node mapping at the time of primary diagnosis and with early detection of recurrence, accrual to future trials of autologous tumor-derived HSPPC-96 will be more limited because of a presumably smaller pool of patients with advanced regional disease.

This trial showed that HSPPC-96 treatment was safe with no unacceptable toxicities or detected autoimmune reactions. A common exclusion criterion in active immunotherapy trials is a negative response to recall antigens by skin testing (Multitest Mérieux; Imtix, Milan, Italy). In the current pilot study, however, we did not exclude anergic patients as we had in earlier whole-cell vaccine trials [15], preferring to remain open-minded regarding an immune response to HSPPC-96 being independent of a DTH reaction. Three of the 5 patients with increasing SFCs by the IFNγ ELISPOT assay had a negative (grade 0-1) DTH response to DNCB at baseline. Nevertheless, future trials should probably exclude anergic patients since anergy is proving to be an active signaling process which can interfere with the induction of an effective systemic cellular immune response [16].

SFC counts against foreign antigens in patients who are exposed to blood borne-infection are generally orders of magnitude higher than those observed against altered self-antigens in patients with malignancy [17]. The relatively weak and transient changes in SFC (overall SFC range for the entire study, 10.7 - 22) during the course of treatment with HSPPC-96 did not show a dose: response relationship. The IFNγ ELISPOT assay may not have been a reliable biomarker, especially since antitumor immune effecter cells which rapidly traffic to the tissue compartment can elude detection by a peripheral blood assay. Alternatively, in the range of doses tested, HSPPC-96 treatment may have been ineffective in mounting a consistent detectable immune response. At the time this study was conducted (1998-2000), not enough fresh material was available to perform the more sensitive tetramer assays.

Among 16 patients with indicator lesions, there was no major objective response, and 81% had clear progression of disease within 8 weeks. Among patients with stage IV disease treated in the adjuvant setting, a median time to progression of 30.4 months with 82% alive at 10 years is encouraging, but it is impossible to determine whether HSPPC-96 treatment contributed directly to this outcome. Immune responses to HSPPC-96 treatment as measured by IFNγ ELISPOT assay were in general weak and transient and correlated poorly with clinical outcomes.

The results presented here can also be compared with those of a subsequent study of HSPPC-96 treatment in metastatic melanoma patients, as reported by Belli et al [9]. Among 28 melanoma patients with indicator lesions there were two reported complete responses, one each at the 5 and 50 μg dose level, lasting >36 and 20 months, respectively. However, there were no partial responses, and the overall median time to progression for the 28 patients was 29 days. There was also no difference in the frequency of immune response by dose level or by route of administration (subcutaneous or intradermal). Eleven patients were treated in the adjuvant setting, and the longest disease-free intervals, reported in 3 patients, were 8, 12, and 21 months, considerably shorter than in the current report.

The progression-free and overall survival data for advanced melanoma patients treated in the adjuvant setting can now be analyzed in context with data from a large phase III trial that included similar patients treated either with Canvaxin, an allogeneic vaccine, or placebo after being rendered surgically free of disease. In that study by Morton et al, 496 patients (out of a planned total enrollment of 670) with stage IV melanoma were treated in the adjuvant setting. The median DFS was 7-8 months with a 5-year DFS of 20.9-27.4 percent. The median overall survival was 32-39 months, with 5-year overall survival of 40%-45% [18]. Although the results of that study do not support the continuation of Canvaxin in clinical development, they do confirm that highly selected stage IV patients who can be rendered surgically free of disease and who remain disease-free long enough after surgery to enroll in stage IV adjuvant cancer vaccine trials can have a prolonged duration of survival. These data contrast the median survival of 8-12 months, with 5-year survival of <5%, for patients who are typically enrolled in stage IV melanoma trials.

The current pilot study excluded patients with evidence of 25% progression of disease in visceral organs or appearance of new disease during the recuperative month after surgery, as these patients were considered to be candidates for other clinical trials at MD Anderson Cancer Center. Had patients with symptomatic or rapidly progressive disease been enrolled, many would have been inevaluable for biomarker response, a primary endpoint of this study. In favoring the recruitment of patients with relatively indolent or no clinical evidence of disease after surgery, the relative contribution of HSPPC-96 treatment to the long term survival of several of the patients with advanced melanoma cannot be ascertained. Furthermore, six of 11 patients with stage IV disease treated with HSPPC-96 in the adjuvant setting may have been favorably affected by prior or subsequent systemic treatment; for example, five of these patients each received at least 6 cycles of biochemotherapy.

Because of the limitations reported in this study, the hypothesis that immunoprotection is best achieved against unique tumor antigens can neither be confirmed nor refuted. The question also remains whether the doses and schedule tested in the current study were adequate to mount an effective immune response. Nonetheless, HSPPC-96 retains conceptual appeal as a tailored active immunotherapy approach.

Persistence of a robust CTL response is likely required to cause tumor shrinkage and this persistence is dependent on continuous antigenic stimulation [19], administration of costimulatory cytokines, inhibition of negative costimulatory molecules (such as cytotoxic T-lymphocyte antigen 4 [20].), and inhibition of immune-suppressing regulatory T cells [21]. A benefit of adding IL2 to HSPPC-96 treatment was not demonstrated in a study by Amato et al. in renal cell carcinoma [22].

There has been substantial advancement in the understanding of the critical attributes of autologous tumor-derived HSPPC-96 as a pharmaceutical product. Increased purity, yield and manufacturing success rate have been achieved through improvements in inhibition of tumor-associated proteases using a protease inhibitor cocktail and in mechanical tissue homogenization. In addition, process steps have been eliminated and chromatographic steps have been optimized. Allowing greater flexibility in the clinic, formal validation studies now support an extended 18-month shelf life, with further improvements expected. (Personal communication, Antigenics, Inc. Lexington, MA).

Kinetics studies are unraveling how peptides which are tightly bound to HSP-96 can nevertheless transfer to MHC class I molecules in an ATP-binding and ATPase dependent step [23,24]. In an effort to limit acquisition of extraneous targets with HSPPC-96, synthetic immunogenic melanoma peptides -- such as MART-1, gp100, or MAGE-- have been bound to cloned HSP-96 and other chaperones, such as HSP-70 [25,26]. Such engineered constructs are being evaluated for their ability to immunize more effectively than peptide alone [27,28].

In summary, despite the lack of demonstrable efficacy of HSPPC-96 as treatment of patient with advanced melanoma in this pilot study, we have demonstrated that clinical-grade HSPPC-96 can be produced from even small amounts of metastatic melanoma tumor tissue and that treatment with HSPPC-96 is safe and well tolerated. HSPs acting as peptide chaperones continue to be an important area of development in the in vivo stimulation of professional APCs against autologous tumor cells.

## Competing interests

The authors declare that they have no competing interests.

## Authors' contributions

OE conceived of the study, participated in its design and coordination, treated and evaluated all the patients, and drafted the manuscript. MR, PM, JL were the surgeons who resected the tumors for autologous vaccine preparation. JL helped with the final preparation of the manuscript. ME coordinated all tumor specimen flow and all protocol requirements on behalf of the patients and did all the in vivo skin testing. CS performed all the ELISPOT and *in vitro *immune assays. NP JE AB were medical oncologists who managed the patients. All authors read and approved the final manuscript.
